# Supplementary material for: LDA2Net Digging under the surface of COVID-19 scientific literature topics via a network-based approach
Source: PLoS One. 2024 Apr 3;19(4):e0300194. doi: 10.1371/journal.pone.0300194 (PMC10990218; doi:10.1371/journal.pone.0300194)
Supplement: S1 File — (PDF) [file pone.0300194.s001.pdf]

Table 7. Human labeling *Vs* automated labeling based on the proposed heuristic. Human labeling has been performed by a medical expert by looking at the list of the top 25 words of each topic only. ND stands for not determined.

Topic #	Human label	2-gram label	3-gram label	4-gram label
1	protective devices	protection→ equipment	protection→ equipment→ used	filtration→ protection→ equipment→ used
2	diagnostics	method→ developed	method→ developed→ used	method→ developed→ rapid→ sensitive
3	statistical analysis (studies)	analysis→ used	data→ analysis→ used	data→ analysis→ used→ analyzed
4	mortality rate related to infection	number→ cases	number→ cases→ deaths	number→ cases→ deaths→ per
5	epidemic models	mathematical→ model	epidemic→ used→ model	reproduction→ number→ R0→ model
6	retrospective clinical study	hospitalized→ confirmed	hospitalized→ confirmed→ without	hospitalized→ confirmed→ without→ diagnosed
7	literature review	various→ aspects	several→ specific→ aspects	aspects→ human→ papillomavirus→ related
8	clinical studies on illness development	mortality→ rate	mortality→ rate→ patients	mortality→ rate→ patients→ risk
9	guidelines for clinical practice management	clinical→ practice	clinical→ practice→ management	clinical→ practice→ management→ regarding
10	statistical analysis (scale assessment)	scale→ scores	scale→ scores→ used	mean→ score→ scale→ scores
11	cellular immune response	inflammatory→ macrophages	inflammatory→ macrophages→ including	inflammatory→ loop→ inflammation→ production
12	online education during pandemic	online→ learning	online→ learning→ students	online→ de→ student→ learning
13	studies on origins	province→ since	province→ city→ china	province→ since→ prevention→ february
14	public health response to pandemics	public→ system	public→ system→ crisis	public→ system→ crisis→ health
15	reviews on recent therapeutical approaches	therapeutic→ approaches	future→ strategies→ various	future→ strategies→ various→ approaches
16	mechanism of cell infection	ACE2→ binding	ACE2→ binding→ expression	receptor→ ACE2→ binding→ expression
17	ND	important→ key	important→ key→ critical	important→ key→ critical→ crucial
18	study of variants	viral→ genome	genome→ sequences→ identified	viral→ genome→ sequencing→ sequence
19	management of surgical procedures	surgical→ procedures	surgical→ procedures→ patients	surgical→ procedures→ patients→ procedure
20	instrumental diagnosis	chest→ CT	chest→ CT→ scan	chest→ CT→ scan→ images
21	contagion diffusion control	contact→ tracing	contact→ tracing→ individuals	contact→ tracing→ persons→ quarantine
22	impact of pandemics on people (social relations)	social→ distancing	social→ distancing→ isolation	social→ distancing→ isolation→ support
23	virus Cov-19 infection	virus→ human	virus→ human→ viruses	SARS→ MERS→ virus→ human
24	pandemics history	help→ us	help→ us→ make	help→ us→ make→ one
25	comparatiove analysis of viremic levels	significantly→ higher	levels→ significantly→ higher	significantly→ higher→ lower→ level
26	access to health of socio-economic groups	household→ income	household→ income→ inequality	population→ derived→ nonprobability→ sample
27	Studies, evidences	evidence→ suggest	evidence→ suggest→ studies	evidence→ suggests→ studies→ needed
28	methodological approaches	approach→ problem	set→ theory→ method	approach→ problem→ time→ algorithm
29	ND	research→ limitations	research→ limitations→ findings	research→ limitations→ development→ process
30	development in India	urban→ population	urban→ population→ area	urban→ population→ planning→ space
31	digital technology	digital→ version	digital→ scanning→ calorimetry	digital→ scanning→ calorimetry→ technology
32	Covid-19 literature	must→ considered	one→ especially→ important	one→ especially→ important→ reasons
33	abstract structure	methods→ results	methods→ results→ study	materials→ methods→ results→ study
34	prevention	prevention→ measures	control→ prevention→ measures	prevention→ measures→ spread→ strategies
35	review studies	systematic→ review	systematic→ reviews→ protocol	systematic→ review→ met→ literature
36	economic impact	tourism→ development	tourism→ development→ business	tourism→ development→ business→ market
37	comparative studies	significantly→ higher	significantly→ higher→ rate	significantly→ higher→ rate→ compared
38	effects of vitamin somministration	levels→ supplementation	levels→ supplementation→ significantly	levels→ supplementation→ significantly→ reduced
39	data	data→ collection	data→ collection→ analysis	data→ collection→ sources→ used
40	publication metadata	without→ use	copyright→ international→ use	copyright→ international→ use→ abstract
41	impact on life quality & sexual health	quality→ life	quality→ life→ expectancy	quality→ life→ expectancy→ health
42	symptoms & clinical manifestations	clinical→ symptoms	gastrointestinal→ infection→ symptoms	clinical→ symptoms→ manifestations→ reported
43	effects of alcohool & addictions	alcohol→ medications	alcohol→ medications→ treatment	alcohol→ medications→ treatment→ cannabis
44	effects on patients with comorbidity	severe→ disease	patients→ severe→ disease	patients→ severe→ disease→ critical
45	impact of air pollution	concentration→ meteorological	concentration→ meteorological→ conditions	concentration→ meteorological→ conditions→ observed
46	correlation with heart disease	heart→ failure	cardiac→ arrest→ patients	heart→ failure→ arrhythmias→ acute
47	ND	new→ york	new→ york→ city	new→ york→ city→ pandemic
48	community	community→ engagement	community→ engagement→ development	sustainable→ strategies→ partnerships→ key
49	stages	early→ especially	early→ especially→ initial	early→ especially→ initial→ results
50	ND	higher→ risk	adjusted→ prevalence→ ratio	adjusted→ prevalence→ ratio→ among
51	age and gender	older→ people	older→ people→ individuals	older→ people→ individuals→ higher
52	interview studies	themes→ emerged	themes→ emerged→ three	themes→ emerged→ three→ key
53	diagnostic tests efficacy	test→ positive	test→ positive→ results	test→ positive→ results→ tests
54	french	de→ patients	de→ la→ santé	de→ la→ santé→ plus
55	pandemic management	pandemic→ response	pandemic→ response→ crisis	pandemic→ response→ crisis→ management
56	nursing programs residents	programs→ program	programs→ program→ value	programs→ program→ value→ virtual
57	effective strategies	effective→ strategies	effective→ strategies→ strategy	effective→ strategies→ strategy→ approach
58	policies for spread containment	control→ measures	containment→ interventions→ implemented	containment→ interventions→ implemented→ reduce

59	technologies	surface→ structure	process→ storage→ efficiency	process→ storage→ parameters→ ph
60	susceptibility to viral infection in epathitys	viral→ infection	viral→ infection→ infections	viral→ infection→ infections→ caused
61	data for the study	analysis→ used	data→ analysis→ used	data→ analysis→ used→ study
62	sociopolitical aspects	political→ cultural	political→ questions→ social	ethical→ issues→ legal→ political
63	cancer therapy	cancer→ treatment	cancer→ treatment→ patients	cancer→ patients→ diagnosis→ therapy
64	pediatry	children→ adolescents	children→ adolescents→ young	children→ adolescents→ young→ families
65	oftalmic	ocular→ surface	eye→ ocular→ surface	ocular→ surface→ conditions→ presence
66	comorbidity diabetes	chronic→ diseases	chronic→ diseases→ disease	chronic→ diseases→ disease→ diabetes
67	rehabilitation after intervention	intervention→ program	intervention→ program→ improve	intervention→ program→ improve→ organizational
68	pandemics in the us	state→ local	state→ local→ federal	state→ local→ federal→ level
69	immunity & specificity of response	igg→ antibodies	igg→ antibodies→ antibody	igg→ antibodies→ antibody→ response
70	online (mis)information	information→ sources	information→ sources→ related	information→ sources→ regarding→ SDM
71	variability	three→ different	different→ patterns→ populations	three→ different→ patterns→ populations
72	healthcare	health→ facilities	health→ facilities→ system	health→ facilities→ system→ planning
73	paper info	web→ response	web→ response→ one	web→ response→ disclosure→ including
74	clinical cases reports	patient→ presented	patient→ presented→ developed	patient→ presented→ developed→ acute
75	numbers	one→ three	one→ three→ study	one→ three→ study→ five
76	UK pandemic timeline	first→ time	first→ time→ UK	first→ time→ UK→ weeks
77	time & duration	median→ time	median→ time→ period	median→ time→ period→ day
78	geographic (countries)	world→ health	around→ world→ health	around→ world→ health→ response
79	impact (on energy market)	paper→ shows	paper→ shows→ impact	paper→ shows→ impact→ examine
80	antiviral treatments	antiviral→ drugs	antiviral→ drugs→ used	antiviral→ drugs→ used→ treatment
81	medical organization and management	emergency→ medicine	medical→ personnel→ emergency	emergency→ medicine→ doctors→ management
82	mental health	psychological→ stress	stress→ disorder→ symptoms	psychological→ stress→ disorder→ symptoms
83	clinical studies on illness development	clinical→ trial	clinical→ trial→ outcomes	clinical→ trial→ outcomes→ registered
84	questionnaire studies	study→ participants	study→ participants→ conducted	study→ participants→ conducted→ aimed
85	damage mechansims	nervous→ system	nervous→ system→ involvement	nervous→ system→ damage→ including
86	anti-inflammatory treatment	patients→ received	patients→ received→ treatment	treatment→ receiving→ tocilizumab→ systemic
87	effects if pandemic	pandemic→ impact	pandemic→ impact→ affected	pandemic→ impact→ affected→ changes
88	antiviral drug molecules	drug→ target	antiviral→ activity→ compounds	antiviral→ targets→ identified→ compounds
89	risk factors	risk→ factors	risk→ factor→ exposure	risk→ factor→ exposure→ factors
90	neurological and cognitive impairment	brain→ including	neurologic→ manifestations→ including	neurologic→ manifestations→ including→ brain
91	respiratory failure	patients→ severe	patients→ severe→ forms	patients→ local→ contestation→ different
92	predictive models	predictive→ value	predictive→ value→ model	predictive→ value→ model→ used
93	behavioural studies	behavior→ intention	positive→ relationship→ perceived	positive→ relationship→ perceived→ behavioral
94	sampling viral rna	viral→ RNA	viral→ RNA→ load	viral→ RNA→ loads→ samples
95	pandemic impact	current→ pandemic	current→ pandemic→ caused	current→ pandemic→ caused→ coronavirus
96	orthopedic traumas	wound→ healing	wound→ healing→ repair	wound→ healing→ repair→ attempt
97	vaccine and immunity	vaccine→ development	vaccine→ development→ efficacy	vaccine→ development→ candidate→ vaccines
98	icu and patinet care	ICU→ patients	care→ unit→ patients	patients→ admitted→ hospitalized→ hospital
99	cellular mechanisms	gene→ expression	gene→ expression→ genes	gene→ expression→ genes→ involved
100	pandemic	coronavirus→ disease	novel→ coronavirus→ disease	coronavirus→ disease→ virus→ spread
101	health care personnel and pandemic	healthcare→ workers	healthcare→ workers→ work	healthcare→ workers→ work→ providers
102	transplant	dental→ treatment	clinical→ course→ dental	clinical→ course→ dental→ treatment
103	structural dynamics	complex→ interactions	complex→ interactions→ structure	complex→ interactions→ structure→ dynamics
104	treatment of cardiovascular complications	stroke→ acute	stroke→ acute→ severe	ischemic→ thrombotic→ outcomes→ complications
105	pandemic in selected countires	italy→ spain	italy→ spain→ one	italy→ spain→ one→ france
106	spanish	de→ la	de→ la→ pandemia	de→ la→ pandemia→ esta
107	AI models	deep→ learning	deep→ learning→ de	deep→ learning→ de→ performance
108	physical activity and exercise	home→ time	home→ time→ confinement	home→ time→ confinement→ le
109	statistics	control→ groups	control→ groups→ significant	control→ groups→ significant→ difference
110	animal models & in vitro	stem→ reduced	stem→ reduced→ lung	stem→ reduced→ lung→ human
111	bacterial resistance to antibacterials	bacterial→ infections	bacterial→ infections→ isolated	bacterial→ pathogens→ identified→ found
112	time studies	total→ number	significant→ increase→ number	ED→ emergency→ visits→ total
113	body weight and metabolism	healthy→ eating	BMI→ obesity→ metabolic	overweight→ BMI→ obesity→ metabolic
114	neo/perinatal infections	included→ among	intrauterine→ transplacental→ flux	intrauterine→ insemination→ doses→ low
115	telemedicine	patient→ care	telemedicine→ visits→ telehealth	telemedicine→ visits→ telehealth→ access
116	coronavirus disease progression	coronavirus→ disease	coronavirus→ disease→ progression	coronavirus→ de→ un→ autre
117	report	reported→ among	reported→ among→ less	reported→ less→ likely→ report
118	severe respiratory syndrome	acute→ respiratory	acute→ respiratory→ syndrome	acute→ respiratory→ syndrome→ coronavirus
119	related works	related→ pandemic	related→ pandemic→ including	status→ related→ pandemic→ including
120	research (publications)	scientific→ research	scientific→ studies→ research	research→ field→ community→ development
