# Supplementary material for: LDA2Net Digging under the surface of COVID-19 scientific literature topics via a network-based approach
Source: PLoS One. 2024 Apr 3;19(4):e0300194. doi: 10.1371/journal.pone.0300194 (PMC10990218; doi:10.1371/journal.pone.0300194)
Supplement: Attachment — Submitted filename: Response_to_Reviewers.pdf [file pone.0300194.s002.pdf]

Response to Reviewers

Giorgia Minello   Carlo R.M.A. Santagiustina   Massimo Warglien

September 1, 2023

To the Editorial Board

Dear Academic Editor,
Dear Reviewers,

Thank you for giving us the opportunity to submit a revised draft of the manuscript titled "`LDA2Net` - *Digging Under the Surface of* COVID-19 scientific literature *Topics via a Network-Based Approach*". We appreciate the time and effort you have dedicated to providing valuable feedback on our manuscript.
In the revised paper, we tried to answer and clarify all questions and remarks. We provided more comprehensive accessibility and references to the underlying data and methods that substantiate the findings presented within the manuscript.
Specifically, in the updated version of the paper, we provide a clearer explanation of the objectives and scopes of our work (in the Introduction and in the Related Work section), a more comprehensive description of the dataset and of the LDA estimation (in the Materials and Methods section and appendix A.2), in particular, concerning the model estimation and selection procedure, and about the model with 120 topics, as suggested by the reviewers.
To clearly demonstrate that the statistical analysis has indeed been conducted properly and rigorously, in addition to changes within the manuscript detailed in the response that follows, other supplementary materials have also been made publicly available online at the following link: https://github.com/carlosantagiustina/underthesurfaceofCOVID19topics/.
Also, we were delighted to seize the opportunity presented by the suggestion of reviewer 1, to compare our results obtained with LDA, to those of other topic models (CTM, STM, etc.), to delimit more clearly the focus of our work and its boundaries.
We believe that thanks to this revision process, the quality of the manuscript now meets the highest publishing standards and hope it fulfills the requirements of PLOS ONE. Notably, we hope the revisions and the additional information provided in the updated version meet your expectations.
Thank you for your consideration. We look forward to hearing from you.
Best regards

The authors

Journal Requirements

1. Please ensure that your manuscript meets PLOS ONE's style requirements, including those for file naming.

   → ok

2. Please note that PLOS ONE has specific guidelines on code sharing for submissions in which author-generated code underpins the findings in the manuscript. In these cases, all author-generated code must be made available without restrictions upon publication of the work.

   → No code sharing.

3. Thank you for stating the following financial disclosure:

   ```
   "C.S. and M.W. acknowledge financial support from the European Union Horizon
   2020 project ISEED (Grant Agreement No. 960366).
   C.S. acknowledges financial support from the European Union Horizon 2020 project MUHAI
   (Grant Agreement No. 951846).
   C.S. acknowledges financial support from PON R&I 2014-2020 (FSE REACT-EU)"
   ```

   Please state what role the funders took in the study. If the funders had no role, please state: ""The funders had no role in study design, data collection and analysis, decision to publish, or preparation of the manuscript.""

   If this statement is not correct you must amend it as needed.

   Please include this amended Role of Funder statement in your cover letter; we will change the online submission form on your behalf.

   → The funders had no role in study design, data collection and analysis, decision to publish, or preparation of the manuscript.

4. Thank you for stating the following in the Acknowledgments Section of your manuscript:

   ```
   "We thank Dr. Martina Gerotto for her 743
   expert support in topic labelling. 744
   C.S. and M.W. acknowledge financial 745
   support from the European Union Horizon 746
   2020 project ISEED (Grant Agreement No. 747
   960366). 748
   C.S. acknowledges financial support 749
   from the European Union Horizon 2020 750
   project MUHAI (Grant Agreement No. 751
   951846)."
   ```

   We note that you have provided funding information that is not currently declared in your Funding Statement. However, funding information should not appear in the Acknowledgments section or other areas of your manuscript. We will only publish funding information present in the Funding Statement section of the online submission form.

   Please remove any funding-related text from the manuscript and let us know how you would like to update your Funding Statement. Currently, your Funding Statement reads as follows:

   ```
   "C.S. and M.W. acknowledge financial support from the European Union Horizon
   2020 project ISEED (Grant Agreement No. 960366).
   C.S. acknowledges financial support from the European Union Horizon 2020 project MUHAI
   (Grant Agreement No. 951846).
   C.S. acknowledges financial support from PON R&I 2014-2020 (FSE REACT-EU)"
   ```

Please include your amended statements within your cover letter; we will change the online submission form on your behalf.

Please remove any funding-related text from the manuscript and let us know how you would like to update your Funding Statement

→ **Funding Statement**
G.M., M.W., and C.S. acknowledge financial support from the European Union Horizon 2020 project ISEED (Grant Agreement No. 960366).
C.S. also acknowledges financial support from from PON R&I 2014–2020 (FSE REACT-EU) and from the EU project MUHAI (Grant Agreement No. 951846).
The funders had no role in relation to the data, content and opinions expressed in the article.

To Reviewers

We thank the reviewers for their critical assessment of our work. In the following, we address their concerns point by point. The reviewers' comments have been listed below in *italic text*, followed by our responses in the standard format. Also, for each comment, if we have modified the manuscript's contents or the appendices, we show changes in a table after each reply, displaying the original and the updated version.

Reviewer 1

Q 1.1 Your references are out-of-date. You should cite up-to-date studies.
- *NET-LDA: a novel topic modeling method based on semantic document similarity*
- *Concept-LDA: Incorporating Babelfy into LDA for aspect extraction*
- *Combining semantic graph and probabilistic topic models for discovering coherent topics*

Reply: We thank the reviewer for this remark. We added the suggested references (Allahyari et al., 2019; Ekinci and Omurca, 2020; Ekinci and İlhan Omurca, 2020) – as well as other recent works that exploit domain knowledge from the semantic web to improve the coherence and interpretability of inferred topics, like Yao et al. (2017) – to the RELATED WORK section of the paper. For the convenience of the reviewer, we show changes in the manuscript here below:

Page & Line	Previous Version	New Version
p. 3 - l. 92	The proposed topic model enrichment method, named `LDA2Net`, allows to incorporate in LDA bigram information without any statistical assumption on the data generation process for word sequences, being based on observed document-level bigram counts.	The proposed topic model enrichment method, named `LDA2Net`, allows to incorporate in LDA bigram information without any statistical assumption on the data generation process for word sequences, being based on observed document-level bigram counts. Differently from other works Yao et al. (2017); Allahyari et al. (2019); Ekinci and Omurca (2020); Ekinci and İlhan Omurca (2020) that aim to improve the coherence and interpretability of inferred topics by using domain knowledge, including Linked Open Data, Knowledge Graph Embeddings, and ontologies from the the semantic web, the topic model enrichment method proposed in this work does not rely on any information or data external to the corpus.

Q 1.2 What is your LDA parameter values?

For example, alpha, beta... There is no information about realization of LDA.

Reply: We thank the reviewer for this suggestion. We have, accordingly, provided further clarifications about the model estimation choices and parameters used in the estimation process in the MATERIALS AND METHODS section and in Appendix A.2. In particular, for what concerns the alpha and beta parameters, both have been estimated by using the *topicmodels* R library, which provides an interface to the C code for LDA models by Blei et al. (2003) and the C++ code for fitting LDA models using Gibbs sampling by Xuan-Hieu Phan et al. (https://gibbslda.sourceforge.net/), we do not impose any constraints on the values of these parameters. The alpha parameter value of the LDA model with 120 topics, estimated using Gibbs Sampling, is 0.4166667, while the beta parameters matrix of size [34563x120], containing the logs of the probabilities of words for each topic, is now available in *.csv* format at the following link: https://drive.google.com/file/d/1awk3UTy7tyJXhKNSve8YQSWdfWQujC4O/view?usp=sharing. In addition, we added a new figure (Fig. 14) displaying the average values of the four criteria used for choosing K.
For the convenience of the reviewer, we show changes in the manuscript and appendices here below:

Page & Line	Previous Version	New Version
p. 4 - l. 134	This section outlines the basic concepts about Latent Dirichlet Allocation (LDA) needed for understanding our approach. As LDA analysis is not the crux of this work, we refer the reader to Blei et al. (2003) and A.1 for a thorough introduction to LDA, and to A.2 for further details about the model estimation and parameters selection.	This section outlines the basic concepts about Latent Dirichlet Allocation (LDA) needed for understanding our approach. As LDA analysis is not the crux of this work, we refer the reader to Blei et al. (2003) and A.1 for a thorough introduction to LDA, and to A.2 for further details about the model estimation and parameters selection. Additional supplementary materials are available online at the following link: `https://github.com/carlosantagiustina/underthesurfaceofCOVID19topics`.
p. 22 - l. 612	To select the number of topics K for the LDA model, we employed the method proposed by Nikita (2016) and the `R` library LDATUNING. For each $K \in \{30, 40, 50, 60, 70, 80, 90, 100, 110, 120, 130, 140\}$ we estimated 5 LDA models using the Gibbs Sampling approach with different seeds and 1300 iterations (with $n.burnin.iter = 300$). Then, four criteria (Arun et al., 2010; Griffiths and Steyvers, 2004; Deveaud et al., 2014; Cao et al., 2009) were employed to choose the optimal value of K. Based on the average values of the considered criteria, $K = 120$ appeared to be the a good candidate number of topics. Finally, we resumed the best run of the LDA estimation obtained for $K = 120$ running 3000 additional iterations to ensure convergence.	To select the number of topics K for the LDA model, we employed the method proposed by Nikita (2016) and the `R` library LDATUNING. For each $K \in \{30, 40, 50, 60, 70, 80, 90, 100, 110, 120, 130, 140\}$ we estimated multiple LDA models with different seeds and 1300 iterations (with $n.burnin.iter = 300$), using the Gibbs Sampling approach. Then, four criteria (Arun et al., 2010; Griffiths and Steyvers, 2004; Deveaud et al., 2014; Cao et al., 2009) were employed to choose the optimal value of K. Based on the average values of the four considered criteria (see Fig. 14), $K = 120$ appeared to be the a good candidate number of topics. Finally, we resumed the best run of the LDA estimation obtained for $K = 120$ running 3000 additional iterations to ensure convergence. The LDA models, and their parameters, have been estimated using the *topicmodels* R library, which interfaces with the C code developed by Blei et al. (2003) and the C++ code for fitting LDA models through Gibbs sampling developed by Xuan-Hieu Phan et al. (`https://gibbslda.sourceforge.net/`). No constraints were imposed on the values for the parameters. For the LDA model with 120 topics, the estimated alpha parameter value is 0.4166667. The beta parameters matrix, of size [34563x120], containing the logarithms of word probabilities for each topic, can be accessed through the following GitHub repository: `https://github.com/carlosantagiustina/underthesurfaceofCOVID19topics`

Q 1.3 You evaluate obtained results in many ways. But beside these, you should evaluate your extracted topics in terms of topic coherence, tf-idf coherence, f-score, precision, recall.
You should compare your proposed topic model with LDA, STM, CTM.

Reply: We thank the reviewer for raising this important point. In order to understand the work as a whole, we would like to stress that the main goal of this project is not the topic modeling *per se*, but to propose a novel way to enrich topic models with bigrams, by using the proposed deterministic technique. In fact, our method can be applied to the outcomes of any estimated topic model (LDA, STM, CTM) that employs a bag-of-words approach (i.e., based on unigrams only), to translate words-by-topic distributions into topic graphs. With this in mind, in this work, we did not compare the performance of different topic models because we aimed at showing the effectiveness of our approach in the topic modeling scope in general. Therefore, we used LDA as a reference tool for any topic modeling, given the fact LDA is the most common approach for this type of task. Nevertheless, in future works, we intend to show how our topic model enrichment technique can be easily applied to other topic model methods, such as correlated topic models (CTM), structural topic models (STM), and others, as suggested by the reviewer, as well as providing a comparative evaluation. As regards the extracted topic evaluation, we did not perform this stage since our project goal is not to measure how interpretable the topics are to humans (the clustering quality) but rather to figure out how our method makes extracted topics more interpretable to humans. Paradoxically, topics with a low coherence metric may only further support our point. Besides, LDA is an unsupervised machine learning algorithm, which means certain accuracy metrics cannot be directly applied since they depend on the notion of true classes for a datum (namely the ground truth, that we do not have).

Page & Line	Previous Version	New Version
p. 3 - l. 101	Even though the method is here applied to a LDA model, it can be implemented with other topic models that can be estimated on words (unigrams), such as CTM Blei and Lafferty (2006) or STM Roberts et al. (2013). One of the critical differences with previously mentioned topic-modeling approaches is that, in this work, the enrichment of the topic model occurs after topics are inferred using unigrams. That means bigrams are later utilized to explore and summarize the syntagmatic structure of topics inferred with LDA at a deeper level.	Even though the method is here applied only to the LDA model, it can be easily implemented with other topic models, such as correlated topic models CTM Blei and Lafferty (2006), or structural topic models STM Roberts et al. (2013). That means our approach can be cast into any topic model framework based on the bag-of-words paradigm to transform the topic's distributions of unigrams into topic graphs. It is worth noting the project's primary focus is not on topic modelling *per se* but enhancing the topic modelling. That is possible through our novel deterministic method, based on bigrams, which takes place after the topic inference stage (i.e., unigrams-by-topic distributions). In other words, bigrams are used to enrich an already estimated topic model and to summarize the syntagmatic structure of inferred topics. For the sake of demonstration, in this work, we will be utilizing only LDA as a basic illustrative example, precisely chosen for its simplicity and widespread use.

Reviewer 2

Q 2.1 The manuscript does not provide additional insightful analysis apart from the topic analysis.
That significantly restricts the contribution of the work. The topic analysis has some degree of randomness due to the initialization, and the outputs are based on statistics. Therefore, the manual interpretation and identification of the topic meanings are essential. Viewing the specific meaning of the target topic, Covid-19 scientific literature, the missing part is very important to distinguish the manuscript from some experiment reports.

Reply: We thank the reviewer for pointing this out. As concerns the additional insightful analysis, we agree this part is missing. Yet, we would like to clarify that our paper's goal is methodological rather than directly related to the medical domain or $COVID-19$. Actually, the choice of the specific case study and corpus was made strategically to serve this methodological purpose. The richness, variety, and complexity of medical literature related to $COVID-19$, made it an ideal (and very demanding) setting to apply the proposed method and see if the results were meaningful from an expert's perspective. We acknowledge that, from a medical application viewpoint, our work does not go beyond a detailed analysis of the network-enriched topics and subtopics identified in the Cord-19 open research dataset but, as we stressed above, the focus of our study is only on developing, exploring and validating the methodology itself. About the randomness of results, a distinction must be made. Our topic enrichment, based on empirical counts of bigrams in each corpus document, does not involve an element of randomness on its own. On the other hand, an element of randomness derives from the estimated topic model (LDA) and its initialization. In other words, the element of randomness is inherited (but not originated by the proposed topic-enrichment method). On the other hand, we also know the LDA algorithm is robust and tends to converge, regardless of the random initialization, so we were not concerned about this matter. In any case, we will better specify and clarify that question in the text. Given that, the randomness aspect might be mitigated, for example, by using non-random topic modelling techniques, such as Structural Topic Modeling with spectral initialization, and that may be an interesting future work. Finally, recalling that the main novelty of this project relies on its original topic-enrichment methodology, we believe that this very facet distinguishes our work from many others based on the same corpus but focusing on medical aspects and related findings.

Page & Line	Previous Version	New Version
p. 2 - l. 34		To better comprehend the ultimate goal of this project, it is essential to stress that the leading idea is not to study the topic modeling *per se* but to introduce a novel way to enhance topic model results and facilitate the interpretation. That in turn means the goal has a methodological character. Actually, the choice of the Cord-19 corpus was made strategically to serve such a purpose. The richness, variety, and complexity of medical literature related to COVID-19, made it an ideal (and very demanding) setting to apply the proposed method and see if the results were meaningful from an expert's perspective. However, it can be exploited for healthcare literature surveying tasks Chen et al. (2023) or bibliometric review Chen et al. (2022) ones.
p. 7 - l. 254		FOOTNOTE It is worth stressing that, as LDA results depend on the algorithm's random initialization, the proposed method also inherits such a randomness aspect. That means different initializations will provide different topics and, in turn by applying our approach different graphs. Yet, given the algorithm's robustness, the results will tend to be very similar.

Q 2.2 The preprocessing method is questionable.

i) The filtering method only removes the articles uploaded before December 31, 2019. Is it a reasonable method? Although the rest of papers are concerning SARS and MERS, are they all related to Covid-19? The inclusive and exclusive criteria of paper filtering are necessary to enhance the trustworthiness of the paper.

ii) You may reference the following papers which focus on literature surveying:

- *Information fusion and artificial intelligence for smart healthcare: a bibliometric study*
- *A bibliometric review of soft computing for recommender systems and sentiment analysis*

Reply: We sincerely appreciate the Reviewer's suggestions for additional references and his thoughtful engagement with the corpus filtration process. Regarding the filtering method applied to the CORD-19 dataset, we opted to remove from the corpus only those articles that were published before December 31, 2019 – the official recognition date of the COVID-19 epidemic by China. This approach was chosen to adopt a conservative stance, ensuring that the articles selected were post-dating the outbreak's formal acknowledgment. While we recognise that some papers published after that data may also address topics such as SARS and MERS, we can assure that the CORD-19 corpus was designed to contain only works related to COVID-19 and SARS-CoV-2. We firmly believe that the creators of the CORD-19 corpus, including the White House medical experts and a coalition of leading research groups, possess a level of expertise in the medical domain that we could not surpass in terms of paper selection. Therefore, the conservative criteria applied through our minimal paper filtering process are precisely aimed at minimising the risks related to

more stringent filtration choice, which could impact the representativeness of the CORD-19 corpus in our work. We are grateful for the opportunity to clarify this point.

Page & Line	Previous Version	New Version
p. 2 - l. 38		Actually, the choice of the Cord-19 corpus was made strategically to serve such a purpose. The richness, variety, and complexity of medical literature related to COVID-19, made it an ideal (and very demanding) setting to apply the proposed method and see if the results were meaningful from an expert's perspective. However, it can be exploited for healthcare literature surveying tasks Chen et al. (2023) or bibliometric review Chen et al. (2022) ones.
p. 4 - l. 123	The dataset contains coronavirus-related research papers from PubMed, bioRxiv, and medRxiv, plus articles from a repository of more than two hundred thousand papers maintained by the World Health Organization. As this work intends to focus on COVID-19, abstracts uploaded before December 31, 2019 (date in which the WHO was first-informed of COVID-related cases of pneumonia in Wuhan) have been filtered out, since mainly concerning SARS and MERS. The resulting dataset contains 398818 abstracts.	The dataset contains coronavirus-related research papers. Papers in the Cord-19 corpus were sourced from PubMed, bioRxiv, and medRxiv, as well as articles from a repository of more than two hundred thousand papers maintained by the World Health Organization. As this work intends to focus on COVID-19, abstracts uploaded before December 31, 2019 (date in which the WHO was first-informed of COVID-related cases of pneumonia in Wuhan) have been filtered out. The resulting dataset contains 398818 abstracts. This conservative filtering approach was adopted for ensuring that the articles selected were post-dating the outbreak's formal acknowledgment and to maintain the representativeness of the CORD-19 corpus in our work.

Q 2.3 Is it correct to claim the paper proposes an automatic topic labeling?
Topic labeling should be the identification and interpretation of the identified topics (word clusters). In my understanding, the paper only discovers the word clusters, but does not provide an automatic method for topic meaning identification. The authors are required to justify this point or revise the manuscript.

Reply: We thank the reviewer for highlighting this issue, and we appreciate the opportunity to clarify our automatic labelling approach. While our method does indeed focus on word cluster identification within topics, it goes beyond mere clustering or grouping of words. Our method enables the discovery of subtopic networks, which are clusters of words with specific association structures that reflect semantic and syntagmatic structures inherent in the corpus. Furthermore, the proposed labelling method generates a frequency-weighted set of label candidates through a statistical random walk technique applicable to weighted and directed graphs, such as our subtopic networks. This automated graph-based approach does not incorporate other information and is clearly not based on semantic AI approaches for reasoning and meaning identification. Instead, it leverages graph properties, like the interconnectedness of words within the reconstructed topic and subtopic networks, to generate label candidates of varying lengths. It is important to note that our method focuses on statistical associations and structural relationships, not semantic or meaning inference. While the method doesn't engage in reasoning, it does provide an automated means of suggesting labels based on

the analyzed structural properties of subtopic networks. We acknowledge the distinction, suggested by the Reviewer, between topic labelling and topic meaning identification, and our method aligns only with the former. We further clarified this aspect in the manuscript to ensure a comprehensive understanding of the approach's limitations and capabilities.

Page & Line	Previous Version	New Version
p. 7 - l. 241		The proposed heuristic does not incorporate information external to the corpus and is not based on semantics. Instead, it leverages graph properties, like the interconnectedness of words within the subtopic networks, to generate label candidates of varying lengths. It is important to note that our method focuses on statistical associations and structural relationships, not meaning inference. While the method doesn't engage in reasoning, it does provide an automated means of suggesting labels based on the analyzed structural properties of word networks.

Q 2.4 I am also curious about the mapping and agreement between the identified topics and the 120 topics in the dataset *(again, are all the 120 topics concerning Covid-19?)*

Reply: We thank the reviewer for highlighting this issue. We wish to clarify that the decision to estimate a topic model with 120 topics is not directly tied to the identification by experts of 120 topics related to Covid-19 within the corpus. Rather, it stems from our methodological choice of using the four criteria proposed by Nikita (2016) to guide the selection of the number of topics in the LDA model we estimate. For each of the 120 topics hence inferred by the LDA model, we engaged a domain expert to provide representative titles/labels that capture the essence of the topic's contents. It is important to note that we did not disclose the automatically generated labels assigned by our method to the topics and subtopics during this process. This was done to ensure that the expert's judgments remained unbiased.

Errata Corrige

Corrections and other minor changes.

Page & Line	Previous Version	New Version
p. 3 - l. 70	However, less attention has been devoted to addressing LDA's limitations and related models concerning their "bag-of-words" approach, neglecting word order. LDA returns a (weighted) list of words for each topic, disregarding the short-distanced semantic information in the word arrangement sequences.	However, less attention has been devoted to addressing the limitations of LDA and related models, concerning their "bag-of-words" approach, which neglect word order. These models return a (weighted) list of words for each topic, disregarding the short-distanced semantic information in the word arrangement sequences.

References

Allahyari, M., Pouriyeh, S., and Kochut, K. (2019). Combining semantic graph and probabilistic topic models for discovering coherent topics. In *Web Intelligence*, volume 17, pages 365–379. IOS Press.

Arun, R., Suresh, V., Madhavan, C. V., and Murthy, M. N. (2010). On finding the natural number of topics with latent dirichlet allocation: Some observations. In *Pacific-Asia conference on knowledge discovery and data mining*, pages 391–402. Springer.

Blei, D. and Lafferty, J. (2006). Correlated topic models. *Advances in neural information processing systems*, 18:147.

Blei, D. M., Ng, A. Y., and Jordan, M. I. (2003). Latent dirichlet allocation. *Journal of machine Learning research*, 3(Jan):993–1022.

Cao, J., Xia, T., Li, J., Zhang, Y., and Tang, S. (2009). A density-based method for adaptive lda model selection. *Neurocomputing*, 72(7-9):1775–1781.

Chen, X., Xie, H., Li, Z., Cheng, G., Leng, M., and Wang, F. L. (2023). Information fusion and artificial intelligence for smart healthcare: a bibliometric study. *Information Processing & Management*, 60(1):103113.

Chen, X., Xie, H., Wang, J., Li, Z., Cheng, G., Wong, M. L., and Wang, F. L. (2022). A bibliometric review of soft computing for recommender systems and sentiment analysis. *IEEE Transactions on Artificial Intelligence*, 3(5):642–656.

Deveaud, R., SanJuan, E., and Bellot, P. (2014). Accurate and effective latent concept modeling for ad hoc information retrieval. *Document numérique*, 17(1):61–84.

Ekinci, E. and İlhan Omurca, S. (2020). Concept-lda: Incorporating babelfy into lda for aspect extraction. *Journal of Information Science*, 46(3):406–418.

Ekinci, E. and Omurca, S. I. (2020). Net-lda: a novel topic modeling method based on semantic document similarity. *Turkish Journal of Electrical Engineering and Computer Sciences*, 28(4):2244–2260.

Griffiths, T. L. and Steyvers, M. (2004). Finding scientific topics. *Proceedings of the National academy of Sciences*, 101(suppl 1):5228–5235.

Nikita, M. (2016). R library: 'ldatuning' version 1.0.2.

Roberts, M. E., Stewart, B. M., Tingley, D., Airoldi, E. M., et al. (2013). The structural topic model and applied social science. In *Advances in neural information processing systems workshop on topic models: computation, application, and evaluation*, volume 4, pages 1–20. Harrahs and Harveys, Lake Tahoe.

Yao, L., Zhang, Y., Wei, B., Jin, Z., Zhang, R., Zhang, Y., and Chen, Q. (2017). Incorporating knowledge graph embeddings into topic modeling. In *Proceedings of the AAAI Conference on Artificial Intelligence*, volume 31.
