# Supplementary material for: LDA2Net Digging under the surface of COVID-19 scientific literature topics via a network-based approach
Source: PLoS One. 2024 Apr 3;19(4):e0300194. doi: 10.1371/journal.pone.0300194 (PMC10990218; doi:10.1371/journal.pone.0300194)
Supplement: Attachment — Submitted filename: Response to Reviewers.pdf [file pone.0300194.s003.pdf]

Response to Reviewers

Giorgia Minello Carlo R.M.A. Santagiustina Massimo Warglien

December 23, 2023

To the Editorial Board

Dear Academic Editor,
Dear Reviewers,

Thank you for giving us the opportunity to submit a revised version of the manuscript "LDA2Net - *Digging Under the Surface of COVID-19 scientific literature Topics via a Network-Based Approach*". We sincerely appreciate the time and attention you have dedicated to our manuscript.
We are happy to learn that both reviewers have noticed the improvements in the overall quality of the paper, and also that they believe that, in the previous revision round, we addressed all their comments.

In the revised work, we tried to answer and clarify the residual concerns of the Reviewers. In particular, we have highlighted in the Response to the the second Reviewer in which sections the details used to obtain the graph in Fig. 1 can be found.
In addition, we seized the opportunity presented by the suggestion of the first Reviewer, to compare results obtained with our enriched LDA model to those that we obtain with enriched versions of other topic models (CTM and SAGE). This to highlight the broad applicability of our enrichment technique. This comparison, which can only be qualitative because there are currently no benchmark techniques for bigram-enriched topic models such as ours, shows that the proposed approach is sufficiently general to be applied to other Probabilistic Graphical Models used for inferring topics in textual corpora, and that, thanks to reconstructed topic networks, our enrichment approach indeed improves the readability of topics, also for other types of models. As anticipated in the previous reply, we intend to work in future works on comparability and benchmark aspects related to the enriched models. In the new Appendix section (F) we provide additional supplementary materials, results and methodological details about the CTM and SAGE topic model estimation and bigrams enrichment, and we visually compare non-enriched and enriched outputs of these two models to those obtained with LDA, which are extensively presented and discussed in the paper.

Supplementary Materials and links to data are publicly available online at the following URL:
https://github.com/carlosantagiustina/underthesurfaceofCOVID19topics/.

We would like to thank the Editor and the Reviewers for guiding us in the review process, and for always providing constructive suggestions and feedback. We hope that, thanks to this process, the quality of the paper now meets the highest publishing standards. Please find below a response to the Reviewers, to address their remarks.
Thank you once again for your consideration. We look forward to hearing from you.

Best regards

The authors

To Reviewers

We thank both reviewers for their assessment of our work. In the following sections, we address the Reviewers' remarks and comments point by point. The Reviewers' comments have been listed below in *italic text*, followed by our responses in the standard format. Also, for each comment, if we have modified the manuscript's contents or the appendices, we show changes in a table after each reply, displaying the updated version.

Reviewer 1

We sincerely thank the first Reviewer for carefully reading our revised paper and the new supplement, and for providing helpful feedback and comments. We hope that new supplementary information provided in the updated version of the Appendix (see section F) will meet your expectations for what concerns the application of our framework to other topic models and their comparison.

Q 1.1 Remark or observation of the reviewer

- *I understand your aim but the comparsion between models was needed in this study. You should compare your proposed topic model with LDA, STM, CTM or any other models.*

Reply: We thank the reviewer for this remark, and we would like to thank him for encouraging us to explore the outcomes that the proposed enrichment method yields when applied to a different topic models, such as the Correlated Topic Model.

To address this remark, we decided to estimate two models, a Correlated Topic Model (CTM) and a Sparse Additive Generative Models (SAGE) model, which we then visually compared using word clouds and topic networks. Results are presented in the Appendix F. Not having considered in our original work covariates that could influence the propensity and content of the themes, to avoid adding further complexity to the comparison, we used decided to use SAGE rather than STM as second model for the comparison. As the Reviewer will notice, thanks to a Spectral initialization, we are able to automatically align topics in the two newly compared models. We would like to stress once again that the enrichment method proposed in our work can be applied to any topic model that has as output a topic-by-document matrix and a feature-by-topic matrix.

For the convenience of the reviewer, we show changes in the manuscript here below (for the new Figures please refer to Appendix F in the manuscript):

Page	New Version
p.21 l.559	For the sake of completeness, we applied our approach to other topic modeling methods, such as the Correlated Topic Model (CTM) and Sparse Additive Generative Model (SAGE), (see Appendix app:comparison), to highlight the broad applicability of our enrichment technique. There are currently no benchmark techniques for bigram-enriched topic models, so the comparison is only qualitative. Yet, we intend to investigate comparability and benchmark aspects related to the enriched models in future works.
p. 27	We compared and applied our approach to three topic modeling methods: LDA, Correlated Topic Model (CTM), and Sparse Additive Generative Models (SAGE). Model implementation parameters are explained in the next section. We employed the same mechanism for both methods, that is, we extracted the bigrams, we performed the topic modeling and then by using the output matrices and the bigram weights we built the graphs for each topic.<br>In order to compare similar topics, we computed the Jaccard Similarity between the top 25 words of each topic. In particular, we selected for the CTM and SAGE models the topics that are closer to topics #50 and #88 obtained with LDA. Results are reported in Table 5. The fact that the LDA topic #50 is close to topic #95 for both SAGE and CTM is no error: it is due to the Spectral initialization technique, which was used for both models. Once found the corresponding topic, to visually inspect and compare topics, we selected the top 30 edges for each network graph. That means we plotted only those nodes (words) belonging to the edge set (given by top 30 bigrams). Comparison are shown in Figures 17 and 18. Clearly, even if they have the same initial node set, the final networks are different as paths built by top edges are not the same.<br>While the networks of enriched CTM and SAGE models are similar in some respects, an outcome we anticipated due to their common initialization, LDA appears so different from the other two that any qualitative comparison of the content becomes complex. We defer to future work for the development of more advanced benchmarking and comparison techniques.
p. 27–29	The input matrices used for the CTM and SAGE models are identical to the ones used for the LDA model presented in the sections above. As for the LDA model, for both CTM and SAGE we set $K = 120$, to facilitate comparability between models. Also, to facilitate comparability between models, we initialized both models using the Spectral initialization technique from the STM R library, as proposed by Lee and Mimno and Lee (2014). Spectral initialization parameters are all set to their default values. This technique allows model outcomes to be deterministic, and hence more easily reproducible and comparable. For both the CTM Blei and Lafferty (2006)and the SAGE Eisenstein et al. (2011) model we use the variational expectation-maximization (VEM) algorithm implemented with the STM library Roberts et al. (2013), we set all parameters to their default values, except for the maximum number of expectation-maximization iterations (max.em.its), that we set to 100. Since both models converge before reaching the aforementioned value, this parameter choice is irrelevant.<br>The CTM converged after 65 iterations (Lower Bound: -286094170). The SAGE model converged after 5 iterations (Lower Bound: -291762273).<br>Using the methods proposed by Roberts et al. (2013) we checked the Beta matrix and the residuals of the CTM and SAGE model and no anomalies / errors have been detected. Since we are interested in visually comparing how different enriched models (LDA vs CTM vs SAGE) compare to each other, we apply to the outputs of both newly estimated models (CTM and SAGE) the bigrams-enrichment technique previously implemented with LDA, as explained in the methods section *From LDA to Topic Networks*. We recall that this enrichment technique is deterministic and independent from the model used for estimating the words-by-topic (TxW) distributions contained in the **M** matrix.

Reviewer 2

We sincerely thank the second reviewer for thoroughly reviewing our revised paper and the additional supplement and offering valuable feedback and comments.

Q 2.1 Remark or observation of the reviewer

- *The authors' responses have covered most of my concerns. The paper could be considered for publication after minor revisions. My concern is about Fig. 1. From the diagram, the graph contains both directional and undirectional edges. But I did not find the contents mentioning the edges' property. Moreover, it will be needed to show how to obtain the graph based on T_n, and how to determine if the edge is directed or undirected.*

Reply: We thank the reviewer for this remark. Yet, we think there may have been a misinterpretation.

Indeed, in Fig. 1, the shown graph (toy-example on the right-hand side of the figure) is directed and weighted (the thickness of the edges, the arrows between nodes, refers to the edge's weight).

We explained in different sections of the manuscript how these topic-specific directed graphs are obtained. For instance in *Network Construction* we wrote "we aim to convert a weighted list of words into a weighted and directed network, a network where each edge has both a direction and weight, and every node has a weight"). In the same section we carefully described how to build a graph starting from the LDA's output matrices as well. In this regard, T_n in Fig. 1 is the list of words of a topic n which is actually derived from LDA's output matrix named $\mathbf{M}$, words-by-topic distributions.

Since all graphs are directed, there is no need to determine whether an edge is directed or not. The direction instead is defined by equation 2 where each bigram order is employed to derive the direction. In particular we stressed at the end of the section that "if the bigram composed by (w_i, w_j) does not exist, it does not imply the bigram $(w_j, wi_)$, in turn, does not exist , i.e. $(w_i, w_j) \neq (w_j, w_i)$. In fact, a bigram represents an oriented edge between two words."

References

Blei, D. and Lafferty, J. (2006). Correlated topic models. *Advances in neural information processing systems*, 18:147.

Eisenstein, J., Ahmed, A., and Xing, E. P. (2011). Sparse additive generative models of text. In *Proceedings of the 28th international conference on machine learning (ICML-11)*, pages 1041–1048.

Mimno, D. and Lee, M. (2014). Low-dimensional embeddings for interpretable anchor-based topic inference. In *Proceedings of the 2014 conference on empirical methods in natural language processing (EMNLP)*, pages 1319–1328.

Roberts, M. E., Stewart, B. M., Tingley, D., Airoldi, E. M., et al. (2013). The structural topic model and applied social science. In *Advances in neural information processing systems workshop on topic models: computation, application, and evaluation*, volume 4, pages 1–20. Harrahs and Harveys, Lake Tahoe.
